# Balanced Fertilization Decreases Environmental Filtering on Soil Bacterial Community Assemblage in North China

**DOI:** 10.3389/fmicb.2017.02376

**Published:** 2017-12-01

**Authors:** Youzhi Feng, Zhiying Guo, Linghao Zhong, Fei Zhao, Jiabao Zhang, Xiangui Lin

**Affiliations:** ^1^State Key Laboratory of Soil and Sustainable Agriculture, Institute of Soil Science, Chinese Academy of Sciences, Nanjing, China; ^2^Department of Chemistry, Pennsylvania State University at Mont Alto, Mont Alto, PA,United States; ^3^Nanjing Institute for Comprehensive Utilization of Wild Plants, Nanjing, China

**Keywords:** fertilization, soil microorganisms, phylogenetic diversity, ecological process, soil fertility

## Abstract

Although increasing evidences have emerged for responses of soil microorganisms to fertilizations, the knowledge regarding community assemblages that cause variations in composition is still lacking, as well as the possible feedback to soil fertility. Phylogenetic conservatism of species indicates their similar environmental preferences and/or function traits and phylogenetic signals further can infer community assemblages and influenced ecological processes. Here, we calculated the mean pairwise phylogenetic distance and nearest relative index, characterizing phylogenetic signal and the undergone ecological process to evaluate the community assembly of soil bacterial phylotypes in 20-year fertilized soils. The bacterial community assembly is structured by environmental filtering, regardless of fertilization regime. Soil phosphorous (P) availability imposes selection on community assemblage and influences their community turnover among fertilizations. When P nutrient lacks, the effect of environmental filtering becomes stronger, hence bacterial functional traits become more coherent; this process results into increased intraspecific interactions characterized by co-occurrence network analysis. In contrast, when P nutrient becomes abundant, the environmental selection is mitigated; function traits are evened. This process reduces intraspecific interactions and increases carbon sequestration efficiency, which is finally of great favor to the increases in soil fertility. This study has made the first attempt, at the bacterial level, to understand how fertilization affects agroecosystems. When more phylogenetic information on how nutrient cycling-related microbes respond to fertilization becomes available, the systematic knowledge will eventually provide guidance to optimal fertilization strategies.

## Introduction

To meet the ever-increasing global demand for foods, the agricultural community continues to search for innovative practices to boost crop yields without sacrificing the environment (Tilman et al., 2011). As one of the key components toward success, our agricultural lands must be fertilized properly. It is now commonly understood that irrational and/or excessive fertilizer application does not always translate into a continuous increase in crop yield (Vitousek et al., 2009). Instead, improperly applied fertilizers often leads to unexpected negative, sometimes severe, environmental consequences, such as non-point source pollution (Ongley et al., 2010). By contrast, a balanced fertilization strategy is better economically and is a more sustainable agricultural practice (Mader et al., 2002; Seufert et al., 2012). Soil microorganisms are essential constituents of the soil ecosystem, and because of extreme sensitivity to environmental changes, the responses of the soil microbial community to fertilization can provide useful understanding of the relationship between fertilization and agroecosystem function and guidance toward more effective fertilization. Therefore, in the past decade, soil scientists have been studying such responses. For example, unbalanced fertilization leads to nutrient deficiency and causes subsequent shifts in the composition of the soil microbial community (Eo and Park, 2016). Long-term imbalances in fertilizations can even adversely affect soil biological health (Bhatt et al., 2016). By contrast, Ling et al. (2014) reported that long-term balanced fertilizations help the soil to build bacterial diversity and enzyme activities. Shen et al. (2010) found that 20 years of organic manure fertilization (OM), a typical balanced fertilization regime, caused shifts in the composition of the soil bacterial community. In previous works, we revealed that 20-year balanced fertilizations increased both soil fertility and crop yields, in addition to shifting the taxonomic composition of the soil bacterial community on the north China plain (Chu et al., 2007). Concomitantly, under OM fertilization, the indigenous *Bacillus* *asahii* became the dominant species (Feng et al., 2015). For functional traits, microcalorimetric analysis revealed the pivotal role of P nutrient in stimulating soil microbial metabolic activity and its influence on soil productivity in soils in north China (Zheng et al., 2009). Despite the increasing number of studies documenting responses of soil microbial diversity and function to fertilization, a systematic understanding of the underlying ecological processes of microbial community assembly and the implications for ecosystem function continue to be lacking.

Because of niche conservatism in phylogeny and niche differences among taxa (Wiens and Graham, 2005; Mayfield and Levine, 2010), hypotheses concerning phylogenetic patterns can lead to inferences about the mechanisms affecting community assembly. Specifically, the phylogenetic conservatism indicates that environmental preferences of bacteria taxa are phylogenetically conserved or that closely related bacteria taxa have the similar function traits. Based on this information, community assemblages and influenced ecological processes can be inferred from their phylogenetic signals. Based on this framework, niche partitioning (through environmental filtering and/or sorting via species interactions) and neutral (or stochastic) processes are classically acknowledged to drive community assembly and are applied to explain diversity patterns in ecosystems (Hubbell, 2001). Analysis of phylogenetic relatedness within communities has been used to evaluate the relative importance of environmental filtering, competitive exclusion, and stochastic processes during community assembly (Webb, 2000; Webb et al., 2002), in addition to network co-occurrence analysis of phylotypes for niche partitioning (Barberan et al., 2012; Dini-Andreote et al., 2014). Although questions have been raised about uses of phylogenies to infer ecological processes involved in community assembly (Silvertown, 2004; Losos, 2008; Mayfield and Levine, 2010; Adler et al., 2013), under the premise that functional traits are phylogenetically conserved, these approaches remain promising tools for disentangling roles in assembly of plant communities (Kembel, 2009; Chase, 2010; Adler et al., 2014; Xing et al., 2014) and microbial communities in a variety of ecosystems (Pontarp et al., 2013; Wang et al., 2013; Dini-Andreote et al., 2014; Liu et al., 2015; Yan et al., 2016). However, to date, to the best of our knowledge, the responses of phylogenetic assemblages of microbial communities to fertilizations in agroecosystems have rarely been examined. In contrast to natural ecosystems, arable soils have relatively homogenous physicochemical properties due to continuous and intensive anthropogenic and land management activities. Therefore, the practices of repeated fertilization and a monoculture system (referred to as continuous cropping or rotation cropping system) likely impose environmental filtering on soil microbial community assembly, based on the selective power of nutrient inputs and crop demand. For the similar reason, our second hypothesis is that balanced fertilization would diminish the effect of environmental filtering on soil microorganisms, because soil fertility increases when balanced fertilization is applied (Mader et al., 2002).

In this study, these approaches were used to increase our understanding of the effects of fertilization on soil ecosystems. The phylogenetic α diversity within communities can be used to disentangle the ecological processes that structure bacterial communities, and the phylogenetic β diversity among communities can reveal the factors that influence phylogenetic community turnover (Graham and Fine, 2008). By analyzing phylogenetic signals of bacterial phylotypes sampled at the Fengqiu Agro-Ecological Experimental Station, established in 1989, we tried to determine the mechanisms of community assembly in response to different fertilizations. Simultaneously, our study revealed possible microbial feedbacks of fertilizations on soil carbon accumulation, which has a vital role in providing essential ecosystem services for humans (Schmidt et al., 2011). These results could increase the general understanding of soil microbial communities in agroecosystems.

## Materials and Methods

### The Long-Term Field Experiment

The long-term field fertilizer experiment was conducted at Fengqiu Agro-Ecological Experimental Station, Chinese Academy of Sciences, Fengqiu County, Henan Province, China (35°00′ N, 114°24′ E). The detailed information is presented in previous papers (Feng et al., 2015). Briefly, seven fertilizations, each with four replicates in completely randomized blocks, were established in 1989. The fertilization treatments were the following: (1) Control, no fertilizer; (2) NK applied as urea and potassium sulfate, respectively, no superphosphate; (3) NP applied as urea and superphosphate, respectively, no potassium sulfate; (4) NPK applied as urea, superphosphate, and potassium sulfate, respectively; (5) OM (organic manure), total N at the same rate as in the NPK treatment but from compost plus chemical P and K fertilizers as in the NPK treatment; (6) OMN, half of N applied as compost and the other half of N and P and K from chemical fertilizers; (7) PK applied as superphosphate and potassium sulfate, respectively, no urea. The crop succession was winter wheat (*Triticum aestivum* L.) and summer maize (*Zea mays* L.), and there was no substantial change in agronomic practice for more than 20 years.

### Soil Sampling

Soil samples were collected in the field after maize harvest in 2012. One composite sample was taken from each quadruplicate plot. Each composite sample (hereafter, sample) was a homogenized mixture of 10 soil cores of 10 cm depth collected using sterile techniques. Each sample was placed in a sterile plastic bag, which was transported to the laboratory at 4°C in 1 week. Subsamples for biological assays were sieved (2 mm mesh) and stored at -20°C for DNA extraction. For chemical assays, soil samples were air-dried and sieved through a 100-mesh sieve to determine soil pH, soil organic carbon (SOC) (Mebius, 1960), total nitrogen (TN) (Nelson and Sommers, 1982), available phosphorus (AP) and available potassium (AK). The microbial biomass C (MC) content was measured using the chloroform fumigation extraction method (Vance et al., 1987). Invertase activity (Ivt) was determined using the 3, 5-dinitrosalicylic acid method as described by Frankenberger and Johanson (1983).

### Soil DNA Extraction

For each soil sample, genomic DNA was extracted from the same amount of moist soil (0.5 g) on the day after sampling using a FastDNA^®^ SPIN Kit for soil (MP Biomedicals, Santa Ana, CA, United States) according to the manufacturer’s instructions. The extracted soil DNA was dissolved in 50 μl of TE buffer, quantified by a spectrophotometer and stored at -20°C until further use. A total of 28 DNA samples were used for high-throughput sequencing analyses.

### Preparation of the Amplicon Libraries for High-Throughput Sequencing

The bacterial community of each soil sample was assayed by high-throughput sequencing. For each DNA sample, the primer set 519F and 907R was used to amplify approximately 400 bp V4–V6 fragments of bacterial 16S rRNA gene (Feng et al., 2015). Briefly, the oligonucleotide sequences included a 5-bp bar code fused to the forward primer as follows: bar code + forward primer. PCR was conducted in 50-μl reaction mixtures with the following components: 4 μl (initial 2.5 mM each) of deoxynucleoside triphosphates, 2 μl (initial 10 mM each) of forward and reverse primers, 2 U of Taq DNA polymerase with 0.4 μl (TaKaRa, Japan), and 1 μl of template containing approximately 50 ng of genomic community DNA as a template. Thirty-five cycles (95°C for 45 s, 56°C for 45 s, and 72°C for 60 s) were performed with a final extension at 72°C for 7 min. The purified bar-coded PCR products from all samples were normalized in equimolar amounts, then prepared using a TruSeq^TM^ DNA Sample Prep LT Kit (Illumina, CA, United States) and sequenced using MiSeq Reagent Kit v2 (500 cycles) following the manufacturer’s protocols. The sequences were deposited in the DDBJ database (accession no. DRA005421).

### Processing of High-Throughput Sequencing Data

Raw sequence data were assembled with FLASH (Magoc and Salzberg, 2011) and processed with the UPARSE algorithm (Edgar, 2013), with primers trimmed with cutadapt (Version 1.9.2) (Martin, 2011). Then, sequences with average quality score below 25 and length less than 300 bp were discarded, and chimeras were filtered by UPARSE. Operational taxonomic units (OTUs) were delineated using a 97% similarity threshold, and taxonomy was determined using the RDP classifier for bacteria (Wang et al., 2007). In total, we obtained 409,690 sequences of bacterial 16S rRNA gene and between 7,707 and 19,950 sequences per sample, with the median value of 14,666 sequences per sample. Because an even depth of sampling is required for alpha (α) and beta (β) diversity comparisons (Shaw et al., 2008), samples were randomly rarified to 7,000 sequences per sample for downstream analysis.

### Phylogenetic Analysis

The species pool was chosen from the input of all species from all fertilizations, for the following reasons: (1) the origin of all fertilized soils was the same soil and (2) the field was not fertilized in the 3 years before the start of the experiment in 1989 to increase soil homogeneity. To evaluate the phylogenetic signal across a range of phylogenetic depths, we used mantel correlograms with 999 randomizations for significance tests (Warren et al., 2008; Diniz-Filho et al., 2010) with the function ‘mantel.correlog’ in the R package Vegan v2.0-2^1^. We partitioned phylogenetic distances into 100 classes, and within each distance class, we tested the correlation coefficient relating between-OTU phylogenetic distances to environmental-optimum distances (Diniz-Filho et al., 2010). This method has the advantage of characterizing shifts in phylogenetic signal across phylogenetic distance classes (Diniz-Filho et al., 2010). Significant positive correlations with the highest coefficient values were at intermediate phylogenetic distances (approximately 45% of the maximum phylogenetic distance) (Supplementary Figure S1), which suggested that microbial functional traits were phylogenetically conserved at intermediate phylogenetic distances. Then, the mean pairwise phylogenetic distance (MPD) and nearest relative index (NRI), the degree of non-random phylogenetic structure of communities, were calculated (Webb et al., 2002; Kembel, 2009). For a single community, when the αNRI value is significantly greater than +2 or less than -2 (two-tailed *t*-test at the 95% confidence level), microbial α diversity assembly is phylogenetically clustered or phylogenetically overdispersed due to environmental filtering or competitive exclusion, respectively (Stegen et al., 2012). By contrast, an αNRI value fluctuating from +2 to -2 indicates that a stochastic process plays a major role in shaping microbial α diversity assembly. The community phylogenetic structure was analyzed using the R package ‘picante’ (Kembel et al., 2010). For the protocol, briefly, αMPD was first calculated to quantify the mean pairwise relatedness of co-occurring OTUs within a community: $\alpha MPD=\Sigma_{i_{k}=1}^{n_{k}}f_{i_{k}}$ mean (Δ*_i_k_j_k__*), where *f_i_k__* is the relative abundance of OTU *i* in community *k*, *n_k_* is the number of OTUs in *k*, and mean (Δ*_i_k_j_k__*) is the mean pairwise phylogenetic distance between OTU *i* and all other OTUs *j* in community *k*. αNRI was then calculated using the ‘ses.mpd’ function (NRI is equivalent to -1-fold the output of ‘ses.mpd’) (Fine and Kembel, 2011) in which the observed αMPD was compared with the null distribution of αMPD generated by 1,000 randomizations of the ‘phylogeny.pool’ null model. For β diversity, βMPD (Fine and Kembel, 2011) and βNRI (Stegen et al., 2015) dissimilarity distances were calculated for phylogenetic community composition comparisons. βNRI is the number of standard deviations that the observed βMPD is from the mean of the null distribution [βNRI = (βMPD_Observed_-mean (βMPD_Null_))/sd (βMPD_Null_)]. βNRI < -2 or >+2 for one pairwise comparison indicates less than or greater than expected phylogenetic turnover, respectively.

### Statistical Analyses

To reveal the variations in the interactions among phylotypes responding to different fertilizations, non-random co-occurrence analysis was conducted for each fertilization regime with four replicates. Briefly, non-random co-occurrence analyses were performed using SparCC (Friedman and Alm, 2012), an approach used to estimate correlation values from compositional data. Initially, co-occurrence patterns were assessed using the checkerboard score (*C*-score) (Stone and Roberts, 1990) in mothur. Then, the 500 most dominant OTUs per fertilization regime were retained for this analysis. For each network analysis, *P*-values were obtained by 9,999 permutations of random selections from the data table. SparCC correlation coefficients (ρ) with an absolute value over 0.85 and statistically significant (*P* < 0.01) were used in network analyses. The nodes in the co-occurrence networks represented the OTUs, whereas the edges corresponded to a strong (ρ-value > +0.85 or <-0.85) and significant (*P* < 0.01) correlation between nodes. To better quantify the topology of networks, a set of network parameters, including numbers of nodes and edges, average path length, network diameter, cumulative degree distribution, clustering coefficient and modularity, among others, were calculated, and networks were visualized using the interactive platform Gephi (Bastian et al., 2009). Proportional to the number of connections is the size of each node.

Statistical procedures were calculated using the IBM Statistical Product and Service Solutions (SPSS) Statistics for windows (Version 13). The data were expressed as the means with standard deviation (SD), and different letters indicated significant differences among different samples. Mean separation among fertilizations was conducted based on Tukey’s multiple range test, following the tests of assumptions of normal distribution, homogeneity of variance and ANOVA. Differences of *P* < 0.05 and *P* < 0.01 were considered significant and highly significant, respectively. Correlations were conducted between richness, MPD and NRI and measured soil parameters, microcalorimetric parameters, network parameters and crop yields.

## Results

### Responses of Bacterial Phylogenetic α Diversity and the Inferred Ecological Processes to Fertilizations

**FIGURE 1:**
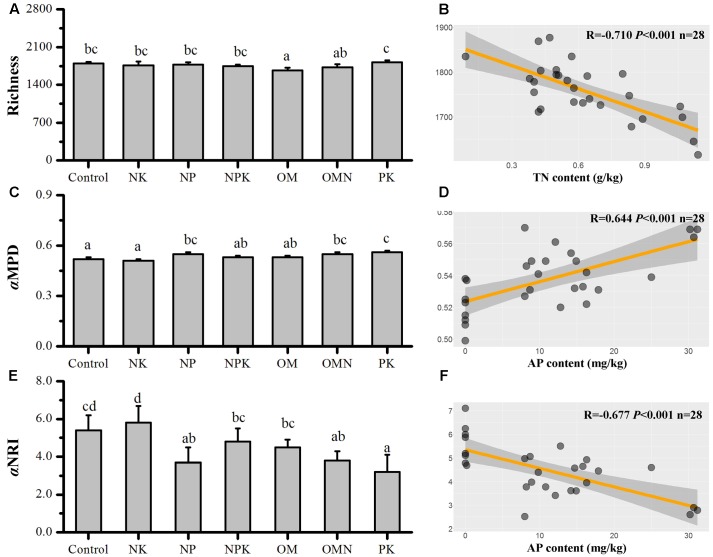
Changes in bacterial Richness **(A)**, αMPD **(C)**, and αNRI **(E)** in response to different fertilizations. And the respective highest correlations (from **Table 1**) to soil chemical factors for Richness **(B)**, αMPD **(D)**, and αNRI **(F)**. Lettering denotes significant differences between fertilizations.

The responses of taxonomic richness values to different fertilizations are presented in **Figure 1A**. The largest difference was for the richness value in OM, which was significantly lower than that in the Control (*P* < 0.05). For the phylogenetic relatedness among soil bacterial phylotypes, Control and NK samples had the lowest αMPD values, which were significantly different from those with high values in NP, OMN, and PK treatments (*P* < 0.05), whereas intermediate values were found in NPK and OM samples (**Figure 1C**). All simulated αMPD values obtained by null model were significantly different from the corresponding observed αMPD values (*P* < 0.01) and all αNRI values obtained using the null model were significantly larger than +2 (*t*-test, *P* < 0.001; **Figure 1E**). These phylogenetic signals indicated that the co-occurring bacterial taxa of all fertilizations were more phylogenetically related than would be expected for a random process and inferred that environmental filtering was operating. The NK treatment clearly had the highest power of environmental filtering on soil microorganisms (*P* < 0.05), followed by the Control, with the lowest effect in the PK treatment.

We performed correlation analyses between different soil chemical parameters (Supplementary Table S1) and richness, αMPD and αNRI (**Table 1**). Richness was significantly positively correlated with pH (*P* < 0.01) and was negatively correlated with SOC, TN (**Figure 1B**), Ivt and MC (*P* < 0.01). αMPD and αNRI were positively and negatively correlated with AP, respectively (*P* < 0.01; **Figures 1D,F**). Additionally, αNRI was negatively correlated with Ivt and αMPD (*P* < 0.05). For the correlations between soil variables, pH was highly significantly negatively correlated with SOC, TN, Ivt, and MC (*P* < 0.01); SOC was significantly positively correlated with TN, AP, Ivt, and MC (*P* < 0.05); TN was significantly positively correlated with AP, Ivt, and MC (*P* < 0.05); and Ivt was significantly positively correlated with AP and MC (*P* < 0.05).

**Table 1:** Pearson correlations between soil properties and Richness, αMPD and αNRI.

	αMPD	αNRI	pH	SOC	TN	AK	AP	Ivt	MC
Richness	0.145	0.007	0.540^∗∗^	-0.663^∗∗^	-0.710^∗∗^	0.099	-0.006	-0.549^∗∗^	-0.486^∗∗^
αMPD		-0.982^∗∗^	0.049	0.080	0.077	-0.11	0.644^∗∗^	0.323	0.136
αNRI			0.055	-0.193	-0.198	0.103	-0.677^∗∗^	-0.421^∗^	-0.217
pH				-0.571^∗∗^	-0.668^∗∗^	0.096	-0.029	-0.508^∗∗^	-0.536^∗∗^
SOC					0.965^∗∗^	-0.171	0.383^∗^	0.898^∗∗^	0.858^∗∗^
TN						-0.131	0.391^∗^	0.862^∗∗^	0.825^∗∗^
AK							0.300	-0.223	-0.211
AP								0.476^∗^	0.336
Ivt									0.758^∗∗^


*SOC, soil organic carbon; TN, total nitrogen; AP, available phosphorus; AK, available potassium; MC, microbial biomass C; Ivt, Invertase activity. ^∗^*P* < 0.05, ^∗∗^*P* < 0.01, respectively. *n* = 28.*

### Responses of Bacterial Phylogenetic β Diversity to Fertilizations

**FIGURE 2:**
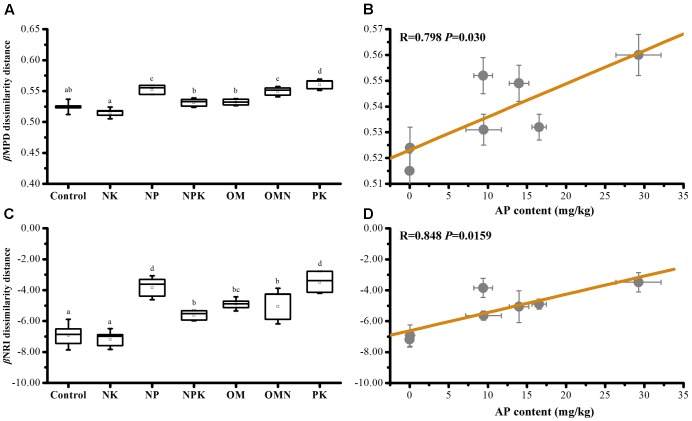
The average bacterial βMPD **(A)** and βNRI **(C)** dissimilarity distances among samples within each fertilization regime, and their significant correlations against AP content in soils **(B,D)**. Lettering denotes significant differences between fertilizations.

Phylogenetic β diversity (the extent of change in community phylogenetic composition, calculated as mean βMPD or βNRI dissimilarity distances within fertilization group (Graham and Fine, 2008), was calculated. Values for βMPD diversity within Control (0.524 ± 0.008) and NK (0.515 ± 0.007) samples were low (*P* < 0.05), whereas values for NP (0.552 ± 0.007), OMN (0.549 ± 0.007), and PK (0.560 ± 0.008) samples were high (*P* < 0.05; **Figure 2A**). A higher β diversity indicates a higher turnover rate between communities or a more heterogeneous composition within one group. In view of the phylogenetic conservatism of the investigated bacterial taxa (Supplementary Figure S1), this phenomenon implied that inferred functional traits were more convergent in P-deficient soils. By contrast, the high phylogenetic β diversity values of NP, NPK, OM, OMN, and PK and the even phylogenetic patterns could be explained by increases in P availability. Concomitantly, dissimilarities in bacterial community composition among fertilizations were observed (Supplementary Figure S2).

The relative influence on community turnover that is determined by homogeneous and variable selection is denoted by βNRI < -2 and βNRI > +2 fractions, respectively (Stegen et al., 2015). For our data, βNRI values were exclusively below -2 (*t*-test, *P* < 0.001; **Figure 2C**), with the inference that homogeneous selection governed community assemblage turnover. Pairwise comparisons further revealed that Control and NK had the lowest βNRI values, NP and PK the highest and the values of NPK, OM, and OMN were intermediate. These phenomena indicated that the environment imposed homogeneous selection on the community assembly in Control and NK treatments to the greatest extent and to the least extent in NP and PK treatments. We also performed statistical analyses on βMPD and βNRI phylogenetic dissimilarity distances among samples within each fertilization regime and against soil chemical properties. Only AP content was significantly correlated with βMPD (**Figure 2B**) and βNRI (**Figure 2D**) values, which indicated that P availability influenced community phylogenetic composition turnover and their congruent traits among fertilized soils.

### Responses of Bacterial Phylotype Co-occurrence to Fertilizations

**FIGURE 3:**
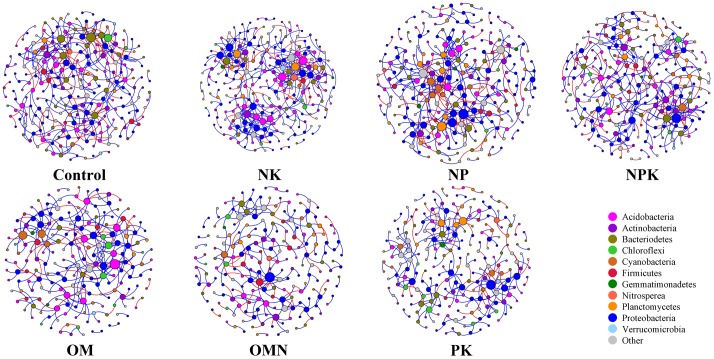
Network co-occurrence analyses of bacterial phylotypes in each fertilization regime. Each node represents a bacterial phylotype (an OTU clustered at 97%). A connection stands for statistically significant (*P* < 0.01) SparCC correlation with a magnitude >+0.85 (positive correlation-blue edges) or <–0.85 (negative correlation-red edges). The size of each node is proportional to the number of connections (that is, degree). Each node is labeled at the phylum level.

We generated co-occurrence networks individually for each fertilization regime (**Figure 3**). The co-occurrence patterns of all networks were non-random (Supplementary Table S2) and differed significantly among fertilizations (PERMANOVA, *F* = 2.4804, *P* = 0.004). The topological parameters are tabulated in **Table 2**. In general, the highest complexities were observed in the communities of Control and NK treatments and the lowest values for those in OM, OMN, and PK. Specifically, all fertilizations led to modularity index values ranging from 0.722 to 0.803. All values were high and above 0.4, a threshold used by Newman (2006), indicating that the partition produced by the modularity algorithm can be used to detect distinct communities within the network. The NK treatment scored several highest values: number of nodes (235) and edges (401), positive (324) and negative (77) correlations, the degree to which they tended to cluster together (average clustering coefficient of 0.060), and node connectivity (average degree of 3.413). In the Control, the set of network parameters were similar to those for NK. By contrast, OM, OMN, and PK had much lower values, and among them, OM had the lowest node number (194), OMN had the lowest edge number (253), and PK had the lowest average degree (2.537).

**Table 2:** Topological properties of networks of each fertilization regime.

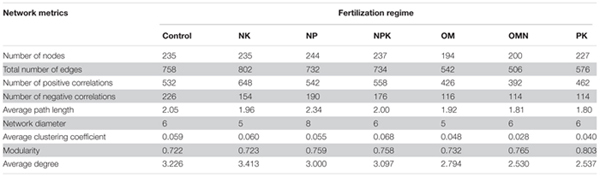

To reveal the mechanisms producing patterns of community coexistence, we analyzed the correlations between co-occurrence network parameters with MPD, NRI and soil properties (Supplementary Table S3). The modularity value was significantly positively correlated with αMPD (*R* = 0.888, *P* = 0.008, *n* = 7), βMPD (*R* = 0.891, *P* = 0.007, *n* = 7) and βNRI (*R* = 0.817, *P* = 0.025, *n* = 7) and significantly negatively correlated with αNRI (*R* = -0.866, *P* = 0.012, *n* = 7). Additionally, average degree was significantly correlated with αMPD (*R* = -0.830, *P* = 0.020), βMPD (*R* = -0.832, *P* = 0.020), αNRI (*R* = 0.872, *P* = 0.011) and βNRI (*R* = -0.774, *P* = 0.041). AP content was significantly positively correlated with modularity (*R* = 0.846, *P* = 0.017) and significantly negatively correlated with average degree (*R* = -0.877, *P* = 0.009) and number of edges (*R* = -0.761, *P* = 0.047). These correlations indicated that high environmental stress and close phylogenetic relatedness, because of lower P availability, could result in more intraspecific interactions within a network.

### Statistical Characterization of Phylogenetic Signals against Soil Microbial Function and Crop Yield under Different Fertilizations

To better study the relationship between phylogenetic signals and functional traits, we analyzed the correlations between richness, αMPD, αNRI, βMPD, and βNRI and the microcalorimetry parameters (Supplementary Table S4) measured in our previous report (Zheng et al., 2009) (**Table 3**). The analysis identified a significant positive correlation between richness and *Q_T_* (total heat dissipation) of soil microorganisms. Additionally, αMPD, βMPD, and βNRI were positively correlated with *P*_max_ (the maximum thermal power) and *K* (soil microbial growth rate constant) (*P* < 0.01) and negatively correlated with *t*_max_ (the time to reach the maximum of the peak) (*P* < 0.05). Of note, the correlations between αNRI and calorimetry parameters (*P*_max_, *t*_max_, and *K*) and those of MPD (α and β) and βNRI were clearly the complete opposite. These phenomena indicated that with the increased influence of environmental filtering, metabolic activities of bacterial taxa decreased simultaneously with more tightly clustered phylogenies.

**Table 3:** Pearson correlations between microcalorimetry parameters and Richness, αMPD and αNRI (*n* = 28), as well as βMPD and βNRI (*n* = 7).

	*P*_max_	*t*_max_	*Q*_T_	*K*
Richness	0.008	0.219	0.471^∗^	-0.049
αMPD	0.725^∗∗^	-0.591^∗∗^	-0.209	0.734^∗∗^
αNRI	-0.744^∗∗^	0.640^∗∗^	0.317	-0.763^∗∗^
βMPD	0.929^∗∗^	-0.770^∗^	-0.342	0.892^∗∗^
βNRI	0.933^∗∗^	-0.873^∗^	-0.565	0.945^∗∗^


*The thermodynamic parameters, that is, growth rate constant (*k*), the maximum thermal power (*P*_*max*_), the time to reach the maximum of the peak (*t*_*max*_), and total heat dissipation (*Q*_*T*_), were obtained by integrating the power-time curves in our previous paper (Supplementary Table S4) (Zheng et al., 2009). ^∗^*P* < 0.05, ^∗∗^*P* < 0.01, respectively.*

**FIGURE 4:**
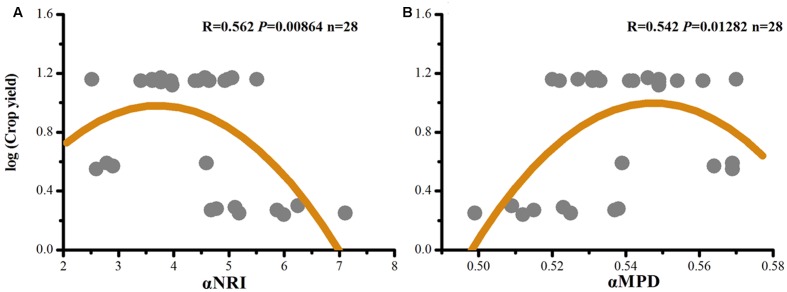
Quadratic correlations of crop yields (Xin et al., 2016) with αNRI **(A)**, and with αMPD **(B)**.

Crop yields ((Xin et al., 2016) (Supplementary Table S5) were correlated with αNRI and αMPD (**Figure 4**). We found a better fit of the data to a quadratic model than to a linear model (αNRI: *R* = -0.429, *P* = 0.023; αMPD: *R* = 0.358, *P* = 0.061). This result indicated the existence of a maximum crop yield due to community assemblage of soil microorganisms; thus, this study can be used to optimize conditions of phylogenetic clustering and environmental stress to maximize crop yield.

## Discussion

### Environmental Filtering Governs Soil Microbial Community Assembly in Agroecosystems

Phylogenetic signals indicated that environmental filtering governed bacterial community assembly regardless of fertilization regime (**Figures 1E**, **2C**). Consistently, all bacterial communities co-occurred more than expected by chance, which is consistent with previous findings (Barberan et al., 2012; Pérez-Valera et al., 2017), suggesting that deterministic processes largely shaped community assembly (Horner-Devine et al., 2007). Although evaluation of phylogenetic patterns can disentangle factors affecting community assembly, the individual effects of environmental filtering and competitive exclusion are sometimes interwoven and not readily separated (Mayfield and Levine, 2010). For example, competitive exclusion can also result in phylogenetic clustering, depending on taxonomic, temporal and spatial scales and species pool (Swenson et al., 2007; Emerson and Gillespie, 2008; Cavender-Bares et al., 2009). However, because of the high conservatism of phylogeny in this study (Supplementary Figure S1), we assumed that environmental filtering was stronger than competitive exclusion in determining community membership in this study. These observations affirmed our first hypothesis, which is also corroborated by Dinnage (2009) who found that plants in disturbed plots are more clustered than expected, whereas ‘undisturbed’ plots are more overdispersed, further demonstrating that biotic communities in disturbed environments are more strongly influenced by environmental filtering. Because assembly based on niche mechanisms is determined by the niche requirements and local habitat conditions (Chase and Myers, 2011), we inferred that the observed niche-based selection of soil microorganisms was largely influenced by interactions among soil physical and chemical characteristics affected by agriculture practices and crop nutrient demands, which shaped the niches and exerted niche forces on the community assembly. Because of the conservatism of species traits in an evolutionary lineage, a positive relationship is found between the phylogenetic relatedness of two species and their overall life history and ecological similarity (Martiny et al., 2015). As a possible explanation, when environmental tolerances of species are phylogenetically conserved, the differences in the environment can act as a filter. As a result, closely related species will be more likely to coexist. For an agroecosystem under intensive anthropogenic activities, such as tillage and plowing, soil physicochemical properties all become relatively more homogenous. Such heavy disturbances create a harsh or unique environment that selects for species that can tolerate these conditions (Warwick and Clarke, 1998; Chase, 2007). Additionally, soil microorganisms function in the belowground part of the crop system and are responsible for providing nutrients to the crop at the expense of a carbon source derived from the crop (i.e., litter and root exudates). Soil microorganisms also experience continual nutrient inputs due to repeated fertilizations. Therefore, these processes also act to filter and structure microbial community assembly. The dynamics of bacterial community succession in a salt marsh chronosequence (Dini-Andreote et al., 2014), with variations in ecological processes, provide supporting evidence (Dini-Andreote et al., 2015). Furthermore, recently, decreases in trophic interactions were found to also lead to phylogenetic (traits) clustering (Pontarp and Petchey, 2016); however, this ecological process was not measured in this study and must be considered in future investigations.

### Soil P Availability Controls the Ecology Process of Microbial Community Assembly in Response to Fertilizations

Although environmental filtering structured bacterial community assembly, the significance of this filtering changed in response to different fertilizations (**Figures 1C,E**, **2A,C**). Overall, the filtering effect was most prominent in P-deficient soils (Control and NK), followed by balanced fertilizations (NPK, OM, and OMN) and P-amended unbalanced fertilization (NP), whereas the effect was lowest in the N-deficient unbalanced fertilization (PK). Our second hypothesis is that the balanced fertilizations would decrease the influence of environmental filtering and was only partly supported by these results. Statistical analyses clearly indicated that AP influenced NRI and MPD (**Figures 1D,F**, **2B,D** and **Table 1**). The situation for intraspecific interactions was also similar (Supplementary Table S3). These results closely support the finding in our previous study for these soils that P is the limiting factor for microbial metabolic activities (Zheng et al., 2009). For soils deficient in P availability (e.g., Control and NK), the agroecosystem imposed a strong stress on microorganisms and only those with similar function traits or environmental preferences could survive, which was depicted by the highest phylogenetic relatedness in P-deficient fertilized soils (**Figures 1C**, **2A**). Moreover, the most complex interactions among taxa were observed in Control and NK treatments (**Figure 3** and **Table 2**), which was a trend that was more significant for the NK treatment. As streamlining theory predicts (Giovannoni et al., 2014), an increase in the competition for resources is always concurrent with increases in cell–cell interactions among microbial members. Consistently, based on the first principles of thermodynamics, Großkopf and Soyer (2016) found that when energy resources are limited in the environment, microorganisms tend to coexist for survival.

By contrast, when *P* was abundant (e.g., NPK, OM, and OMN; Supplementary Table S1), the environmental stress was alleviated (**Figure 1F**). For the ecological processes, Liu et al. (2015) reported that with an increase in soil nutrients, the effect of environmental filtering on arbuscular mycorrhizal fungal (AMF) community assembly decreased. Additionally, an increase in soil fertility introduces more stochastic effects/processes in the microbial community to offset the influence of niche partitioning (Chase, 2010; Zhou et al., 2014; Deng et al., 2016). Dini-Andreote et al. (2014) found the complexity of a bacterial phylotype network decreases in more productive soils. In our study, the findings were similar, and compared with the Control and NK treatment, the absolute values of α and βNRI of balanced fertilized soils decreased (**Figures 1E**, **2C**), and they had simple network patterns (**Figure 3** and **Table 2**). Additionally, because the nutrient K was abundant in the soils (Supplementary Table S1) and not the limiting factor for crop and soil microorganisms, NP could be considered a balanced fertilization, and therefore, the environmental filtering in that treatment also decreased, compared with the Control and NK treatment (**Figures 1E**, **3** and **Table 2**). As a further notable observation, the lowest environmental stress was in PK with lowest αNRI values, which was accompanied by the simplest network pattern. Although N is the predominant element for crop growth, why N deficiency did not impose the greatest environmental filtering on community assembly remains unexplained. A possible explanation could be that a deficiency in N is not as limiting to soil microorganisms, compared with P (Supplementary Table S4). Similarly, Xu et al. (2016) found that P influences tree species community assembly, whereas N has no effect. Additionally, when crop growth is limited by the availability of N (Supplementary Table S5), more P in soils could be available for microbial growth (Supplementary Table S1). Thus, for these reasons, the lowest influence of environmental filtering was observed for the soil community assembly in the PK treatment. However, further investigation is required to verify this speculation.

### Phylogenetic Signal Feedback to Soil Fertility

As an important component of agricultural soil, SOC determines the fertility and sustainability of arable lands (Gregorich et al., 1994; Jimenez et al., 2002). Soil microorganisms play important roles in the transformation of organic matter and SOC accumulation (Trumbore and Czimczik, 2008). Thus, their phylogenetic signals can be a useful tool in studying the intrinsic microbial gap between fertilization and soil fertility. In previous investigations, we found that soils with balanced fertilizations typically have higher carbon sequestration efficiency than that of P-deficient treated soils (Zheng et al., 2009). More recently, we found that P-deficiency decreases the retention of exogenous labile C in soil through increases in soil microbial respiration (Jing et al., 2017). Therefore, under P-deficient fertilizations, a significant amount of C is readily lost as CO_2_ through respiration, resulting in a lower percentage of C retained as biomass in soil. A possible explanation is that P-deficiency causes a strong environmental stress on soil microorganisms, which subsequently boosts the growth of soil microbial community members specialized in P acquisition for the crop. With the increase in growth, much of the carbon source is consumed to provide energy for nutrient cycling. A study of AMF exemplifies such a scenario (Lin et al., 2012). In Control and NK fertilized soils, crops require a diverse AMF assemblage with a large population size to meet the demands for growth. As a reciprocal reward, the plant increases the carbohydrate supply to AMF (Kiers et al., 2011). Consequently, because plants are the primary source of carbon to soils, carbon and energy resources available to other soil microorganisms likely decrease. The slow microbial growth that was accompanied by a large unit cell heat output observed in P-deficient soils was an example of this effect (Supplementary Table S4) (Zheng et al., 2009). Moreover, the most complex patterns of co-occurrences of bacterial phylotype networks were found for Control and NK soils in our study (**Figure 3** and **Table 2**). Such high interspecies dependency due to nutrient limitation (Giovannoni et al., 2014) may lead to high C consumption. All the abovementioned processes are adverse for SOC sequestration, soil fertility and crop yield (**Figure 4** and Supplementary Table S6).

By contrast, in a nutrient-rich environment, plants can uptake sufficient nutrients directly from soil without requiring microorganisms specialized in P acquisition. More carbon and energy are then available to stimulate the growth of other soil microorganisms (Supplementary Table S4). The expectation is that the accelerated growth and the possible underlying evolutionary process will result in microorganisms with more functional traits, which is exemplified by the even phylogenetic relatedness in soils with balanced fertilizations (**Figures 1C**, **2A**). A diversification of functional traits of soil microorganisms is favorable to the stability and sustainability of arable soils (Rolland et al., 2012; Bluthgen et al., 2016). Because microbial function is highly correlated with carbon sequestration efficiency (Zheng et al., 2009; Jing et al., 2017), the natural inference is that the underlying mechanism of balanced fertilizations is better soil sequestration of carbon (Liang et al., 2016).

Although soil fertility and crop yield generally increase with a decrease in level of environmental stress, in our study, crop yield began to decrease in the treatment with the least environmental stress (e.g., PK; **Figure 4**). The implication is that a maximum crop yield occurs under an optimal condition of phylogenetic clustering and environmental stress. In a natural ecosystem, diverse functional traits of soil microorganisms are required to implement ecosystem multifunctionality to resist exogenous interferences (Soliveres et al., 2016); however, the situation is different in an agroecosystem. Only relatively coherent functional traits, supplemented by human agricultural practices, are required for the maximization of crop yield. Thus, overdispersed functional traits due to less environmental stress are expected to reduce, instead of to increase, the efficiency of soil microorganisms for nutrient cycling reactions in arable soils. Therefore, we assumed that only a proper environmental stress (i.e., fertilization strategy) could direct soil microorganisms to the maximum productivity. However, such a speculation requires further tests, particularly by those employing a big data approach.

## Conclusion

In a strategically designed, long-term fertilization field study, the phylogenetic signal of the bacterial community and the co-occurrence network changed in response to different fertilization regimes. Based on analyses of those changes, we revealed the underlying ecological processes driving variations and determined that environmental filtering structured bacterial community assembly. An increase in soil P availability, under P-amended fertilizations, reduced the extent of environmental filtering on soil microorganisms, and as result, functional traits became more evenly dispersed and the complexities of the co-occurrence networks were simplified. Through these processes, soil carbon sequestration greatly increases as a result of more diversified microbe activities, which eventually leads to increases in soil fertility. To our best knowledge, this study is the first attempt to investigate the ecological process that correlates soil microbial phylogenetic signals and assemblages in response to fertilizations. It is worthwhile to note that only bacteria taxa were evaluated in this study, and our findings were derived from the dominant taxonomical species only. More microbial phylogenetic information (such as that of fungi, of functional groups involved in C and P sequestration, and with higher taxonomical resolution in response to fertilization strategies) is definitely needed for a systematic knowledge. When such comprehensive microbial information is available, we could eventually apply the conceptual understanding to the development of more sustainable and economical agricultural practices.

## Author Contributions

XL and JZ designed the study. YF and FZ performed the experiments. YF, ZG, and XL analyzed the data. YF, LZ, ZG, and XL wrote the paper.

## Conflict of Interest Statement

The authors declare that the research was conducted in the absence of any commercial or financial relationships that could be construed as a potential conflict of interest.

## Footnotes

1 http://vegan.r-forge.r-project.org
