# Supplementary material for: Balanced Fertilization Decreases Environmental Filtering on Soil Bacterial Community Assemblage in North China
Source: Front Microbiol. 2017 Dec 1;8:2376. doi: 10.3389/fmicb.2017.02376 (PMC5716987; doi:10.3389/fmicb.2017.02376)
Supplement: Supplementary file 1 [file Data_Sheet_1.DOCX]

**Supplementary information**

**Table S1** The chemical properties of different fertilized soils

| **Treatment** | **pH** | **SOC** | **TN** | **AK** | **AP** | **Ivt** | **MC** |
| --- | --- | --- | --- | --- | --- | --- | --- |
| Control | 8.87±0.10 | 7.65±0.81 | 0.33±0.16 | 81.30±4.81 | 0.05±0.10 | 10.72±2.40 | 78.32±10.71 |
| NK | 8.72±0.13 | 8.01±0.95 | 0.42±0.01 | 347.21±12.64 | 0.00±0.00 | 10.54±1.76 | 45.11±0.59 |
| NP | 8.68±0.06 | 10.46±0.29 | 0.57±0.01 | 64.09±1.91 | 9.39±1.18 | 21.07±2.65 | 71.75±16.86 |
| NPK | 8.65±0.12 | 11.34±0.74 | 0.65±0.04 | 175.04±16.04 | 9.45±2.27 | 23.91±4.36 | 117.60±22.06 |
| OM | 8.60±0.09 | 18.98±0.4 | 1.10±0.04 | 177.91±2.21 | 16.55±0.9 | 34.53±3.82 | 200.19±46.03 |
| OMN | 8.63±0.06 | 14.2±0.47 | 0.84±0.03 | 155.91±6.53 | 13.97±1.25 | 27.07±4.64 | 157.80±34.93 |
| PK | 8.83±0.05 | 9.38±0.40 | 0.50±0.01 | 326.17±10.99 | 29.26±2.89 | 17.88±4.62 | 84.15±15.56 |

**Table S2** Checkerboard score (C-score) of co-occurrence patterns within each fertilization regime

| Treatment | C-Score | *P* value |
| --- | --- | --- |
| Control | 0.4418 | <0.01 |
| NK | 0.4400 | <0.01 |
| NP | 0.4443 | <0.01 |
| NPK | 0.4483 | <0.01 |
| OM | 0.4430 | <0.01 |
| OMN | 0.4442 | <0.01 |
| PK | 0.4399 | <0.01 |

**Table S3** Pearson correlations between phylogenetic signal, soil chemical properties and co-occurrence network parameters (n=7)

|  | ***α*MPD** | ***α*NRI** | ***β*MPD** | ***β*NRI** | **pH** | **SOC** | **TN** | **AK** | **AP** |
| --- | --- | --- | --- | --- | --- | --- | --- | --- | --- |
| **Number of nodes** | -0.117 | 0.227 | -0.122 | -0.160 | 0.521 | -0.851* | -0.836* | -0.046 | -0.401 |
| **Total number of edges** | -0.593 | 0.671 | -0.596 | -0.581 | 0.369 | -0.728 | -0.732 | -0.041 | -0.761* |
| **Number of positive correlations** | -0.609 | 0.671 | -0.606 | -0.559 | 0.279 | -0.691 | -0.677 | 0.226 | -0.669 |
| **Number of negative correlations** | -0.391 | 0.479 | -0.404 | -0.457 | 0.443 | -0.593 | -0.633 | -0.563 | -0.726 |
| **Average path length** | -0.053 | 0.083 | -0.056 | 0.035 | -0.007 | -0.245 | -0.261 | -0.624 | -0.470 |
| **Network diameter** | 0.585 | -0.522 | 0.580 | 0.518 | 0.071 | -0.254 | -0.235 | -0.602 | 0.037 |
| **Average clustering coefficient** | -0.642 | 0.661 | -0.643 | -0.493 | 0.143 | -0.399 | -0.413 | -0.099 | -0.610 |
| **Modularity** | 0.888** | -0.866* | 0.891** | 0.817* | 0.085 | -0.021 | 0.034 | 0.235 | 0.846* |
| **Average degree** | -0.830* | 0.872* | -0.832* | -0.774* | 0.207 | -0.516 | -0.535 | -0.026 | -0.877** |

**Table S4** Results from power-time curves measured by microcalorimetric method in the report of Zheng et al. [^1^](#_ENREF_1)

| **Treatment** | ***P*_max_ (μW)** | ***t*_max_ (min)** | ***Q*_T_ (J g^-1^)** | ***K* (min^-1^)** |
| --- | --- | --- | --- | --- |
| Control | 259.73(5.55)B | 1353.50(40.99)C | 12.89(0.88)D | 0.0049(0.0009)A |
| NK | 198.35(21.70)A | 1515.25(40.13)D | 12.08(3.57)CD | 0.0031(0.0005)A |
| NP | 627.58(93.29)DE | 694.88(120.16)B | 9.66(0.61)BC | 0.0111(0.0038)B |
| NPK | 538.87(35.66)CD | 700.75(14.72)B | 8.83(0.09)AB | 0.0090(0.0027)B |
| OM | 478.28(14.78)C | 577.38(89.37)A | 7.74(0.16)A | 0.0088(0.0011)B |
| OMN | 611.87(96.08)DE | 674.25(53.98)AB | 9.71(2.08)BC | 0.0094(0.0001)B |
| PK | 679.88(85.97)E | 583.50(83.58)A | 10.61(0.49)C | 0.0107(0.0006)B |

**Table S5** Crop yields of 2012 recorded by Xin et al. [^2^](#_ENREF_2)

| **Treatment** | **Grain yield* (t/hm^2^)** |
| --- | --- |
| Control | 1906d |
| NK | 1814d |
| NP | 13963b |
| NPK | 14644a |
| OM | 13860b |
| OMN | 14460ab |
| PK | 3757c |

*Grain yield is the oven-dry yields of summer maize and winter wheat in 2012.

**Table S6** Pearson correlations between microcalorimetry parameters and network parameters (n=7)

| **Correlations** | **Number of nodes** | **Total number of edges** | **Number of positive correlations** | **Number of negative correlations** | **Average path length** | **Network diameter** | **Average clustering coefficient** | **Modularity** | **Average degree** |
| --- | --- | --- | --- | --- | --- | --- | --- | --- | --- |
| *P*_max_ | -0.198 | -0.626 | -0.621 | -0.456 | -0.065 | 0.515 | -0.520 | 0.867* | -0.823* |
| *t*_max_ | 0.422 | 0.726 | 0.717 | 0.536 | 0.128 | -0.300 | 0.459 | -0.691 | 0.814* |
| *Q*_T_ | 0.514 | 0.562 | 0.508 | 0.507 | 0.041 | -0.046 | 0.185 | -0.268 | 0.495 |
| *K* | -0.179 | -0.581 | -0.607 | -0.361 | 0.078 | 0.572 | -0.425 | 0.776* | -0.763* |


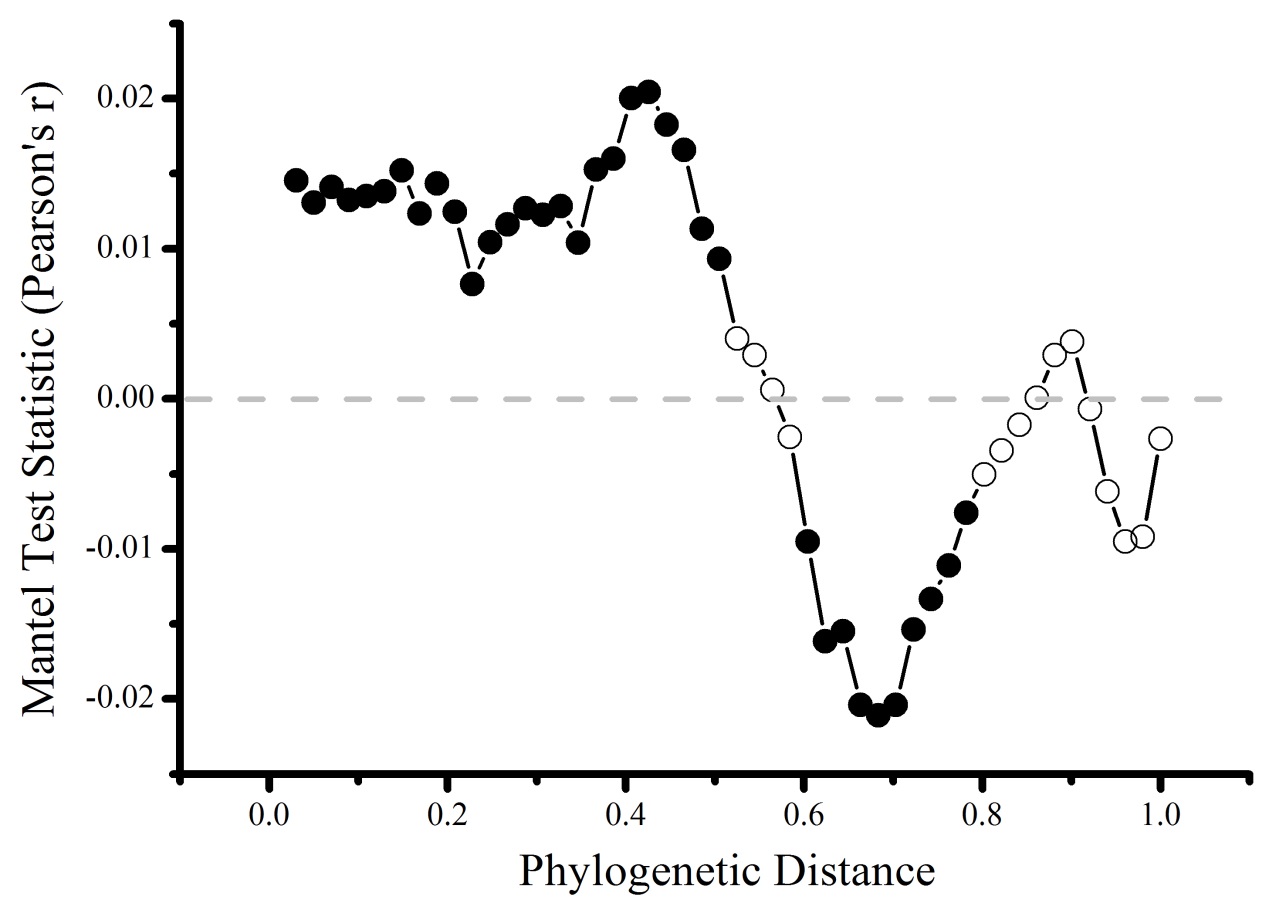


**Figure S1.** Phylogenetic Mantel correlogram showing significant phylogenetic signal across intermediate phylogenetic distances. Solid and open symbols denote significant and nonsignificant correlations, respectively, relating between-OTU niche differences to between-OTU phylogenetic distances across a given phylogenetic distance.


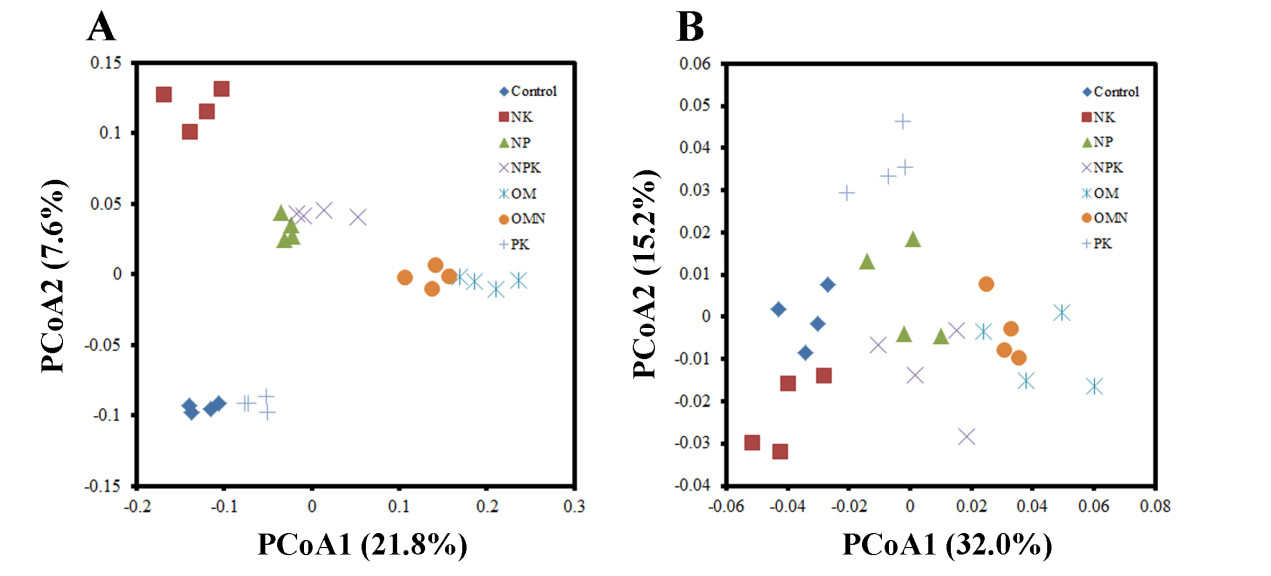


**Figure S2.** The shifts in bacterial community composition in response to fertilizations, indicated by principal coordinate analysis (PCoA) plot of the Bray Curtis (A) and weighted Unifrac (B) dissimilarity between soil samples based on 7,000 sequences per soil sample.

The PCoA plots of Bray Curtis (Fig. S2A) and Unifrac (Fig. S2B) dissimilarity clearly demonstrated significant overall bacterial composition variability among fertilizations. Specifically, points for Control and NK soil samples, lacking exogenous P amendment, cluster in the left-most side of the figure. P amendments help to move data points for NP, PK and NPK to the middle section. When soils are treated with organic manure, OMN and OM data appear on the right-most side in the figure. Such clustering/separations are confirmed by ANOSIM analysis on Bray Curtis distance-based dissimilarity: the group of Control and NK (*R*=0.523, *P*=0.001), the group of OM and OMN (*R*=0.722, *P*=0.001) and the group of NP, NPK and PK (*R*=0.172, *P*=0.012).

**References**

1. Zheng, S. X.; Hu, J. L.; Chen, K.; Yao, J.; Yu, Z. N.; Lin, X. G. Soil microbial activity measured by microcalorimetry in response to long-term fertilization regimes and available phosphorous on heat evolution. *Soil Biol. Biochem.* **2009,** *41* (10), 2094-2099.

2. Xin, X. L.; Zhang, J. B.; Zhu, A. N.; Zhang, C. Z. Effects of long-term (23 years) mineral fertilizer and compost application on physical properties of fluvo-aquic soil in the North China Plain. *Soil Till. Res.* **2016,** *156*, 166-172.
